# Association between serum non-high-density lipoprotein cholesterol and cognitive impairment in patients with acute ischemic stroke

**DOI:** 10.1186/s12883-016-0668-2

**Published:** 2016-08-26

**Authors:** Da Lu, Pan Li, Yuying Zhou, Xiaolin Xu, Huihong Zhang, Liping Liu, Zhiyan Tian

**Affiliations:** 1 Department of Neurology, Sixteenth wards, Tianjin Huanhu Hospital, Tianjin, 300060 China; 2 Department of Neurology, Second wards, Tianjin Huanhu Hospital, Tianjin, 300060 China

**Keywords:** Dyslipidemia, Non-high-density lipoprotein cholesterol, Ischemic stroke, Cognitive disorders

## Abstract

**Background:**

Non-high density lipoprotein cholesterol (HDL-C) could be a good predictor of vascular disease outcomes. To evaluate the association between serum non-HDL-C and cognitive impairment in patients with acute ischemic stroke.

**Methods:**

A total of 725 hospitalized patients with acute ischemic stroke were enrolled. They received conventional treatment. Cognitive function was assessed on the 3rd day after admission using mini-mental state examination (MMSE), Montreal Cognitive Assessment (MoCA), Activity of Daily Living Scale (ADL), and Neuropsychiatric Inventory (NPI, and Hamilton depression rating scale 21-item (HAMD-21). Lipid profile and biochemical markers were measured, and non-HDL-C was calculated.

**Results:**

Compared with patients with normal non-HDL-C, those with high non-HDL-C showed lower MMSE (23.1 ± 4.9 vs. 26.0 ± 4.6, *P* < 0.001) and MoCA (20.4 ± 6.4 vs. 22.2 ± 5.3 *P* = 0.01) scores, higher NPI (6.2 ± 1.2 vs. 3.3 ± 1.5, *P* < 0.001) and HADM-21 (6.0 ± 2.2 vs. 4.5 ± 1.9, *P* < 0.001) scores, and higher homocysteine (16.0 ± 3.8 vs. 14.3 ± 2.0 mmol/L, *P* = 0.044), fasting blood glucose (6.4 ± 2.7 vs. 6.1 ± 2.1 mmol/L, *P* = 0.041), and HbA1c (6.80 ± 1.32 % vs. 6.52 ± 1.17 %, *P* = 0.013) levels. MMSE (*r* = -0.526, *P* < 0.001), MoCA (*r* = −0.216, *P* < 0.001), and NPI (*r* = 0.403, *P* < 0.001) scores were correlated with non-HDL-C levels. High non-HDL-C levels were an independent risk factor for cognitive disorders after acute ischemic stroke (*P* = 0.034, odds ratio = 3.115, 95 % confidence interval: 1.088–8.917).

**Conclusions:**

High serum non-HDL-C levels, age, education, homocysteine levels, and HAMD score were independent risk factors of cognitive impairment in patients with acute ischemic stroke. The risk of cognitive disorders after acute ischemic stroke increased with increasing non-HDL-C levels. This parameter is easy to assess in the clinical setting.

## Background

Acute ischemic stroke is an episode of neurological dysfunction caused by cerebral ischemia persisting for >24 h or until death [1], and represent 80–87 % of all strokes [2, 3]. In high income countries, the incidence of stroke is 217 per 100,000 person-years and the prevalence is 715 per 100,000 people, compared with low income countries where the incidence is 281 per 100,000 person-years and the prevalence is 393 per 100,000 people. Likely risk factors include history of transient ischemic attacks, cardiovascular diseases (hypertension, myocardial infarction), smoking, metabolic syndrome, heavy alcohol consumption, type 2 diabetes mellitus, high cholesterol levels, carotid artery stenosis, genetic factors, and biochemical factors [2, 3].

Vascular cognitive impairment (VCI) is one of the most common non-somatic manifestations in patients with acute ischemic stroke [4]. Dyslipidemia is not only an independent and maybe the most important risk factor for ischemic stroke, which tends to cause hidden, progressive, and somatic organic damage, but also affects the cognitive function of patients with ischemic stroke through accelerating systemic atherosclerosis, and it is believed to be a risk factor inducing cognitive disorders, even dementia [5].

Previous guidelines for blood lipid treatment preferred low-density lipoprotein cholesterol (LDL-C) as the primary target of lipid-lowering therapy [6]. However, some recent studies and epidemiological investigations indicated that serum non-high-density lipoprotein cholesterol (HDL-C) levels were superior to LDL-C in terms of predicting the risk of cardiovascular diseases. The National lipid association recommendations for patient-centered management of dyslipidemia issued by the American National Lipid Association (NLA) in 2015 proposes that non-HDL-C is preferable to LDL-C as the primary target of interventions [7], since non-HDL-C includes all lipoprotein particles other than the HDLs, therefore including the triglyceride-rich lipoproteins [chylomicrons, very-low-density lipoproteins (VLDL), and their remnants], which are now known to participate in atherosclerosis [7]. Nevertheless, the exact relationship between non-HDL-C and cognitive impairment after stroke is still poorly understood.

Therefore, this study aimed to investigate the association between serum non-HDL-C levels and cognitive function after stroke, as well as the value of non-HDL-C levels in assessing the risk of cognitive disorders after acute ischemic stroke.

## Methods

### Study design

This was a retrospective case-control study of patients with acute ischemic stroke hospitalized and registered at the Department of Neurology of the Tianjin Huanhu Hospital between January 2010 and December 2015. The study was approved by the ethical committee of the Tianjin Huanhu Hospital. The need for individual consent was waived by the committee because of the retrospective nature of the study.

### Patients

Inclusion criteria were: 1) newly diagnosed with ischemic stroke according to the China cerebral vascular disease guidelines [8] based on computed tomography (CT) and/or magnetic resonance imaging (MRI); 2) onset time ≤7 days; and 3) aged >18 years old. Exclusion criteria were: 1) disturbance of consciousness, severe aphasia, or hemiplegia, and unable to complete neuropsychological testing; 2) Alzheimer’s disease (AD), depression, dementia with Lewy bodies (DLB), frontotemporal dementia (FTD), or dementia caused by other diseases such as malignant tumors, intracranial infection, neurodegenerative diseases, craniocerebral trauma, etc.; 3) heart, lung, liver, kidney, or endocrine system diseases, or connective tissue diseases, blood disease, or malnutrition; 4) history of mental diseases or behavior disorders; 5) history of ischemic stroke with cognitive disorders based on the medical files or inquiry to the family at admission; or 6) history of nootropics or antipsychotic drugs within 4 weeks.

### Data collection

Patients’ gender, age, height, body mass index (BMI), level of education, family history of dementia, presence of vascular risk factors such as hypertension, diabetes, hyperlipidemia, heart diseases (atrial fibrillation, myocardial infarction, angina pectoris, asystole, etc.), history of stroke and/or transient ischemic attack (TIA), smoking history, and drinking history were recorded in details at admission.

### Grouping

The patients were divided into three subgroups according to the Oxfordshire Community Stroke Project (OCSP) classification [9]: 1) total/partial anterior circulation infarction (TACI/PACI) group; 2) posterior circulation infarction (POCI) group; and 3) lacunar circulation infarction (LACI) group. TACI manifests as a triad, i.e. manifestations of complete middle cerebral artery syndrome: 1) cerebral and high nervous disorders (disturbance of consciousness, aphasia, acalculia, spatial disorientation, etc.); 2) homonymous hemianopia or conjugate eye deviation; and 3) motor and/or sensory disturbance at three contralateral parts (face, upper limbs, and lower limbs). PACI manifests as two out of three manifestations. POCI manifests as various degrees of vertebro-basilar artery syndromes: 1) ipsilateral cerebral palsy and contralateral sensory disorders; 2) bilateral sensory and motor disorders; 3) collaborative movement disorders of eyes and cerebellar dysfunction, which are unequal brain-stem and cerebellar infarctions caused by obstruction of vertebro-basilar artery and branches. LACI manifests as lacunar syndrome.

### Biochemistry

Overnight fasting blood samples were routinely drawn. Fasting blood glucose, total cholesterol (TC), triglycerides (TG), HDL-C, and LDL-C were measured using an ADVIA-2400 automatic biochemical analyzer (Siemens, Erlangen, Germany) and the triglyceride test kit (GPO-PAP, Shanghai Huachen, Shanghai, China). A BNP II protein analyzer (Siemens, Erlangen, Germany) was used to test serum high-sensitivity CRP (hs-CRP) (C-reactive protein kit, Siemens Healthcare Diagnostics Products GmbH, Erlangen, Germany), glycated hemoglobin (HbA1c) (HBIC HbA1c kit, Siemens Healthcare Diagnostics Products GmbH, Erlangen, Germany), and homocysteine (Hcy) (Hcy kit, Siemens Healthcare Diagnostics Products GmbH, Erlangen, Germany).

Non-HDL-C was calculated by subtracting HDL-C levels from TC. Non-HDL-C levels were classified into two grades according to the criteria recommended by the *Patient-centered management of dyslipidemia* developed by the NLA in 2015 [7]: normal group (non-HDL-C <3.4 mmol/l) and high group (non-HDL-C ≥3.4 mmol/l). The high group can be further divided into higher than normal (3.4 mmol/l ≤ non-HDL-C <4.2 mmol/l), critical high (4.2 mmol/l ≤ non-HDL-C <5.0 mmol/l), and very high (non-HDL-C ≥5.0 mmol/l).

### Neurological assessment

After admission, patients were routinely given anti-platelet, anticoagulation, expansion, and other conventional treatments for stroke [3]. On the 3rd day after admission, their neuropsychological scales were routinely assessed by trained and skilled neurologists. Cognitive function was screened using the mini-mental state examination (MMSE) [10] and the Montreal Cognitive Assessment (MoCA) [11] scales. The total score of MMSE is 30 points, of which <17 points, <20 points, and <24 points in illiterate patients, patients with education level of primary school, and patients with education level of secondary school or above, respectively, are considered as cognitive disorders. The total score of MoCA is 30 points, where a score <26 points is considered as cognitive disorders, using a score +1 to correct for education level for patients with ≤12 years of education. MoCA presents a higher sensitivity in screening mild cognitive disorders, while MMSE is more effective and convenient for dementia patients with multiple-domain impairment. In this study, results of the above two scales were comprehensively considered since they were only used as quantitative indices of cognitive level variation with the change of non-HDL-C.

Neuropsychological behavior was assessed using the neuropsychiatric inventory (NPI) [12] to determine the frequency and severity of behavioral disorders as well as distress. The NPI included 12 items and a total score of 144 points, where a higher score refers to more severe psychological and behavioral disorders.

The activities of daily living (ADL) were assessed using the ADL scale [13], which includes 20 items: 11 items for basic ADL (BADL) and 9 items of instrumental ADL (IADL), for a total score of 80 points, where a higher score refers to a more severe impairment.

The emotional state was assessed using the Hamilton Rating Scale 21-item HAMD-21 [14], where a score ≥7, ≥17, and ≥24 points refers to mild, moderate, and severe depression, respectively.

### Statistical analysis

Statistical analysis was performed using SPSS 19.0 (IBM, Armonk, NY, USA). Normally-distributed continuous data are presented as mean ± standard deviation and were analyzed using the independent-samples *t*-test or ANOVA with the LSD post hoc test, as appropriate. Categorical data are expressed as frequency and were analyzed using the chi-square test. Correlations were assessed using the Pearson correlation test. Logistic regression (stepwise) was performed to determine the factors independently associated with higher cognitive impairment. Two-sided *P*-values <0.05 were considered statistically significant.

## Results

### Characteristics of the patients

A total of 725 patients with acute cerebral infarction were hospitalized at the Department of Neurology of the Tianjin Huanhu Hospital between October 2010 and March 2015. They were divided into two groups according to the non-HDL-C levels: the normal group included 253 patients (192 men and 61 women, aged 54–85 years, mean age of 63.1 ± 11.9 years) and the high group included 472 patients (336 men and 136 women, aged 52–81 years, mean age of 62.2 ± 10.8 years). Furthermore, each group was further divided into three subgroups according to the OCSP classification: TACI/PACI, POCI, and LACI.

Gender, age, years of education, as well as hypertension, diabetes, coronary atherosclerotic heart disease, smoking history, drinking history, family history of dementia, and stroke history were similar between the two groups (*P* > 0.05) (Table 1), but BMI in the normal non-HDL-C group was significantly lower than in the high non-HDL-C group (*P* = 0.009) (Table 1). TC, TG, HDL-C, LDL-C, HCY, fasting blood glucose, HbA1c, and other indicators were statistically different between the two groups (*P* < 0.05) (Table 1), except for hsCRP (*P* > 0.05). There was no significant difference in OCSP subtypes between the two groups (*P* > 0.05).

**Table 1:** Socio-demographic and clinical characteristics of the patients

Variables	Normal non-HDL-C (*n* = 253)	High non-HDL-C (*n* = 472)	*P*
Gender, n (%)			
M	192 (75.9)	336 (71.2)	0.175
F	61 (24.1)	136 (28.8)	
Age (years)	63.1 ± 11.9	62.2 ± 10.8	0.289
BMI (kg/m^2^)	24.6 ± 3.4	25.3 ± 3.3	0.009
Years of education (years)	9.2 ± 4.3	9.1 ± 3.9	0.788
Hypertension, n (%)	167 (66.0)	317 (67.2)	0.753
Diabetes, n (%)	58 (22.9)	101 (21.4)	0.636
Coronary atherosclerotic heart disease, n (%)	43 (17.0)	88 (18.6)	0.583
Smoking history, n (%)	90 (35.6)	168 (35.6)	0.996
Drinking history, n (%)	67 (26.5)	122 (25.9)	0.853
Family history of dementia, n (%)	10 (4.0)	19 (4.0)	0.962
Stroke history, n (%)	94 (37.2)	151 (32.0)	0.161
Non-HDL-C (mmol/l)	2.77 ± 0.46	4.40 ± 0.86	<0.001
TC (mmol/l)	3.86 ± 0.52	5.54 ± 0.88	<0.001
TG (mmol/l)	1.27 ± 0.56	1.95 ± 0.31	<0.001
HDL-C (mmol/l)	1.08 ± 0.26	1.13 ± 0.25	0.027
LDL-C (mmol/l)	2.16 ± 0.46	3.39 ± 0.80	<0.001
HCY (mmol/l)	14.28 ± 2.01	16.02 ± 3.80	0.044
hsCRP (mg/l)	4.35 ± 0.78	4.47 ± 1.97	0.895
Fasting glucose (mmol/l)	6.05 ± 2.06	6.44 ± 2.66	0.041
HbA1c (%)	6.52 ± 1.17	6.80 ± 1.32	0.013
TACI/PACI, n (%)	167 (66.0)	309 (65.5)	0.264
POCI, n (%)	67 (26.5)	111 (23.5)	
LACI, n (%)	19 (7.5)	52 (11.0)	

*BMI* body mass index, *non-HDL-C* non-high-density lipoprotein cholesterol, *TC* total cholesterol, *TG* triglycerides, *HDL-C* high-density lipoprotein cholesterol, *LDL-C* low-density lipoprotein cholesterol, *HCY* homocysteine, *hsCRP* high-sensitivity C-reactive protein, *HbA1c* glycated hemoglobin, *TACI* total anterior circulation infarction, *PACI* partial anterior circulation infarction, *POCI* posterior circulation infarction, *LACI* lacunar circulation infarction

### Impact of non-HDL-C levels on cognitive impairment

The MMSE and MOCA scores in the high non-HDL-C group were significantly lower than in the normal non-HDL-C group (MMSE: *P* < 0.001; MoCA: *P* = 0.01). Assessments of neuropsychological behavior and emotion showed that the NPI and HADM-21 scores in the high non-HDL-C group were higher than in the normal non-HDL-C group (*P* < 0.001). Meanwhile, the ADL scale scores were similar between the two groups (*P* > 0.05) (Table 2).

**Table 2:** Comparison of neuropsychological scale between the two groups

	Normal non-HDL-C (*n* = 253)	High non-HDL-C (*n* = 472)	*P*
MMSE	26.0 ± 4.6	23.1 ± 4.9	<0.001
MoCA	22.2 ± 5.3	20.4 ± 6.4	0.010
NPI	3.3 ± 1.5	6.2 ± 1.2	<0.001
HADM-21	4.5 ± 1.9	6.0 ± 2.2	<0.001
ADL	25.7 ± 8.0	28.2 ± 4.1	0.230

*MMSE* mini-mental state examination, *MoCA* Montreal Cognitive Assessment, *NPI* Neuropsychiatric Inventory, *ADL* Activity of Daily Living Scale, *HADM-21* Hamilton depression rating scale 21-item

### Association of non-HDL-C levels and neurological scores

The high non-HDL-C group was further divided into the higher than normal non-HDL-C (208 cases), critical high non-HDL-C (183 cases), and very high non-HDL-C (81 cases). The NIHSS was significantly higher in patients with critical high non-HDL-C levels compared with patients with higher than normal levels (*P* < 0.05)(Table 3).

**Table 3:** Comparison of the non-HDL-C differences according to neurological scores

	Higher than normal non-HDL-C levels (208 cases)	Critical high non-HDL-C levels (183 cases)	Very high non-HDL-C levels (81 cases)	*P*
NIHSS	4.0 ± 1.3	3.6 ± 1.1^a^	4.8 ± 1.8	0.043
NPI	3.9 ± 1.2	4.1 ± 1.2	4.9 ± 1.5^ab^	0.021
BI	84.1 ± 14.6	84.8 ± 14.4	82.9 ± 16.1	0.217
MMSE	24.9 ± 4.6	24.6 ± 5.1	22.6 ± 6.0^ab^	<0.001
MoCA	20.5 ± 5.0	20.6 ± 5.9	18.3 ± 4.3^ab^	0.002
ADL	26.3 ± 8.2	26.5 ± 8.4	29.2 ± 10.3	0.450
HAMD	4.8 ± 1.3	4.7 ± 1.0	4.2 ± 2.7	0.634

*NIHSS* NIH stroke score, *MMSE* mini-mental state examination, *MoCA* Montreal Cognitive Assessment, *NPI*, Neuropsychiatric Inventory, *ADL* Activity of Daily Living Scalem, *HADM-21* Hamilton depression rating scale 21-item

^a^ANOVA and LSD post hoc test: *P* < 0.05 vs. the higher than normal non-HDL-C group

^b^ANOVA and LSD post hoc test: *P* < 0.05 vs. the critical high non-HDL-C group

### Correlations of non-HDL-C level with various indices among patients with high non-HDL-C levels

Pearson correlation analyses revealed that MMSE (weak correlation, *r* = -0.237, *P* < 0.001) and MoCA (weak correlation, *r* = −0.194, *P* < 0.001) scores were negatively correlated with non-HDL-C level in patients with acute ischemic stroke. In order to correct the impacts of gender, age, education level, disease history, and other confounding factors, we further performed partial correlation analyses, which showed that MMSE (moderate correlation, *r* = −0.526, *P* < 0.001) and MoCA (weak correlation, *r* = −0.216, *P* < 0.001) scores were negatively correlated with non-HDL-C levels, while NPI scores (weak correlation, *r* = 0.301, *P* < 0.001) were positively correlated with non-HDL-C levels (Table 4).

**Table 4:** Correlation of non-HDL-C levels with neurological scores

Items	Pearson correlation analysis		Partial correlation analysis	
	*r*	*P*	*r*	*P*
MMSE	−0.237	<0.001	−0.526	<0.001
NPI	0.059	0.2	0.301	<0.001
MoCA	−0.194	<0.001	−0.216	<0.001
HAMD-21	0.028	0.494	0.046	0.316
ADL	0.036	0.388	0.047	0.311

The partial correlation analysis is corrected for the impact of gender, age, education level, and disease history

*MMSE* mini-mental state examination, *MoCA* Montreal Cognitive Assessment, *NPI* Neuropsychiatric Inventory, *ADL* Activity of Daily Living Scale, *HADM-21* Hamilton depression rating scale 21-item

### Effects of stroke site on cognitive function

Assessment of cognitive function showed that MMSE and MoCA scores were significantly different among different subtypes of cerebral infarction in the two groups (*P* < 0.05), of which the score was the lowest in the TACI/PACI group, followed by the POCI and LACI groups. In addition, among different cerebral infarction subtypes, the MMSE and MoCA scores were significantly lower in the high level group compared with the normal level group (*P* < 0.001) (Table 5).

**Table 5:** Comparison of MMSE and MoCA scores of patients with different cerebral infarctions between the two groups

	Normal level (*n* = 253)	High level (*n* = 472)	*P*
MMSE			
TACI/PACI	28.49 ± 1.19	21.94 ± 5.13	<0.001
POCI	29.01 ± 0.83	22.47 ± 4.22	<0.001
LACI	29.50 ± 0.51	23.98 ± 4.04	<0.001
*P*	<0.001	0.019	
MoCA			
TACI/PACI	25.10 ± 2.97	17.29 ± 6.50	<0.001
POCI	26.05 ± 2.68	17.95 ± 5.68	<0.001
LACI	27.35 ± 1.93	19.62 ± 5.72	<0.001
*P*	0.003	0.045	

*MMSE* mini-mental state examination, *MoCA* Montreal Cognitive Assessment, *TACI/PACI* total/partial anterior circulation infarction, *POCI* posterior circulation infarction, *LACI* lacunar circulation infarction

### Risk analysis of non-HDL-C levels to cognitive impairment after ischemic stroke

Univariable analyses revealed that non-HDL-C levels, education level, age, history of diabetes, history of stroke, family history of dementia, HAMD score, and HCY level (*P* < 0.05) were associated with cognitive disorders after acute ischemic stroke (Table 6). These factors were included in the multivariable analysis and the results showed that high non-HDL-C levels, history of stroke, low education level, age, HAMD score, and HCY levels were independently associated with cognitive disorders after ischemic stroke (all *P* < 0.05).

**Table 6:** Univariable and multivariable analyses of factors associated with cognitive disorders after acute ischemic stroke

	Univariable			Multivariable		
Variables	*P*	OR	95 % CI	*P*	OR	95 % CI
Gender, male	0.239	0.729	0.431–1.234			
Age	0.001	1.043	1.018–1.068	0.001	1.039	1.016–1.064
BMI	0.623	0.982	0.913–1.056			
Years of education	<0.001	0.817	0.772–0.866	<0.001	0.808	0.757–0.861
Hypertension	0.324	1.207	0.831–1.755			
Diabetes	<0.001	1.276	1.175–1.385	0.341	1.400	0.700–2.797
Coronary heart disease	0.388	1.380	0.664–2.870			
History of smoking	0.367	1.226	0.788–1.907			
History of drinking	0.524	1.221	0.661–2.254			
Family history of dementia	0.001	1.219	1.082–1.374	0.055	0.242	0.057–1.031
History of stroke	0.021	1.742	1.087–2.791	0.025	1.723	1.071–2.771
High TC	0.629	0.791	0.306–2.044			
High TG	0.674	1.078	0.760–1.529			
Low HDL-C	0.088	0.650	0.396–1.066			
High LDL-C	0.900	1.025	0.694–1.514			
High Non-HDL-C	0.034	3.115	1.088–8.917	<0.001	3.115	1.088–8.917
Normal level	0.170	1.691	0.799–3.579	0.088	1.691	0.799–3.579
Higher than normal	0.046	2.710	1.017–7.221	0.052	2.710	1.017–7.221
Critical high level	0.026	5.877	1.231–28.065	0.040	5.877	1.231–28.065
Very high level	0.013	7.006	1.502–32.675	0.932	7.006	1.502–32.675
High Hcy	<0.001	1.060	1.032–1.088	<0.001	1.057	1.031–1.084
hsCRP	0.515	0.990	0.960–1.021			
Fasting hyperglycemia [n (%)]	0.285	1.084	0.935–1.256			
High HbA1c	0.960	1.002	0.939–1.068			
NPI	0.218	1.039	0.978–1.104			
HAMD-21	0.001	1.224	1.089–1.375	0.001	1.284	1.177–1.401
ADL	0.141	1.012	0.996–1.029			

*BMI* body mass index, *Non-HDL-C* non-high-density lipoprotein cholesterol, *TC* total cholesterol, *TG* triglycerides, *HDL-C* high-density lipoprotein cholesterol, *LDL-C* low-density lipoprotein cholesterol, *HCY* homocysteine, *hsCRP* serum high-sensitivity C-reactive protein, *HbA1c* glycated hemoglobin, *MMSE* mini-mental state examination, *MoCA* Montreal Cognitive Assessment, *NPI* Neuropsychiatric Inventory, *ADL* Activity of Daily Living Scale, *HADM-21* Hamilton depression rating scale 21-item

## Discussion

Non-HDL-C could be a good predictor of vascular disease outcomes. Therefore, this study aimed to evaluate the association between serum non-HDL-C and cognitive impairment in patients with acute ischemic stroke. Results showed that compared with normal non-HDL-C, patients with high non-HDL-C showed lower MMSE and MoCA scores, higher NPI and HADM-21 scores, and higher HCY, fasting blood glucose, and HbA1c levels. MMSE, MoCA, and NPI scores were correlated with non-HDL-C levels. High non-HDL-C levels were an independent risk factor of cognitive disorders after acute ischemic stroke.

Non-HDL-C was initially used to predict the risk of cardiovascular diseases and is a strong predictor for all-cause mortality and cardiovascular disease mortality, and is significantly superior to LDL-C levels [15]. The Bypass Angioplasty Revascularization Investigation (BARI) study assessed the efficacy of secondary prevention in 1514 patients with multiple coronary diseases, and their 5-year follow-up revealed that non-HDL-C levels were an independent predictor for non-fetal myocardial infarction, while LDL-C levels failed to have significant predictive effect on the primary endpoint or mortality [16]. In another clinical longitudinal study with a follow-up >19 years in 2406 men and 2058 women, the risk of coronary disease in men with non-HDL-C levels >220 mg/dl was 2.14 times that of men with non-HDL-C levels <160 mg/dl, while that in people with LDL-C levels >190 mg/dl was 1.77 times that of people with LDL-C levels <130 mg/dl [15]. Recent studies proposed that serum non-HDL-C levels were closely associated with cerebral vascular diseases. Indeed, Wu et al. [17, 18] conducted a follow-up study of 95,916 18-98 year-old patients without stroke or myocardial infarction in Tangshan (Hebei, China) and found that serum non-HDL-C levels were an independent risk factor of ischemic stroke, and that high serum non-HDL-C levels were associated with an increase of >50 % of the risk of ischemic stroke in the healthy population, which was better than the association between LDL-C and the same risk. In their study, 13.0 % of the patients had asymptomatic intracranial arterial stenosis (ICAS) [17, 18]. In addition, they found that the elevation extent of serum non-HDL-C levels was positively correlated with ICAS incidence, and was an independent risk factor for it (OR = 1.15) [19]. Patients with mild cognitive impairment (MCI) caused by intracranial stenosis are prone to deteriorate and progress to dementia, and its mechanism might be because atherosclerosis cause vascular distortion and enwinding, but also might induce mechanical occlusion of intracranial vessels. In addition, an important atherosclerotic stenosis might indicate systemic atherosclerosis and extensive microvascular diseases, damage of microcirculation, increased resistance of small vessels and decreased vascular reactivity, ultimately leading to cerebral hypoperfusion. The cumulative effect of these interdependent hemodynamic disorders might play a key role in accelerating the onset and progression of cognitive disorders [20]. Some studies also found that serum non-HDL-C levels were significantly increased in patients with MCI compared with people with normal cognitive function, and the cognitive score was negatively correlated with the non-HDL-C levels (*r* = −0.761) [21]. This cross-sectional analysis first discovered that the non-HDL-C levels in 65.1 % of the patients with acute ischemic stroke was significantly higher than the normal value, and that it was positively correlated with the prevalence and damage extent of cognitive impairment after acute ischemic stroke. Thus, serum non-HDL-C level might have high value in predicting the disease since it was an independent risk factor of vascular cognitive impairment.

In this study, various neurological scores were assessed in patients with different levels of serum non-HDL-C, and patients with serum non-HDL-C levels higher than the normal value tended to present significant cognitive impairment and were accompanied by apparent mental behavior and emotion disorders, significantly affecting their ability and quality of life. Multivariable regression analyses found that the risk of cognitive disorders after acute ischemic stroke was increased significantly when non-HDL-C levels were higher than normal. Therefore, serum non-HDL-C levels were an independent risk factor of cognitive disorders after cerebral stroke. A recent study showed that high LDL-C levels and low HDL-C levels were associated with β-amyloid-associated cognitive impairment, suggesting that cholesterol levels play an important role in cognitive function [22]. Additional studies are necessary to assess their precise role in neurodegenerative diseases and cognitive impairment.

Damage from non-HDL-C to cognitive impairment after stroke could be associated with its strong association with the development of atherosclerosis. First, non-HDL-C particles include all potentially atherogenic lipoproteins [23–25]. Secondly, a high concentration of TG and VLDL-C in non-HDL-C reflects an increase of the liver’s potential to generate lipoproteins causing atherosclerosis, leading to decreased interaction of these lipoproteins with liver receptors and reduced clearance. Therefore, these lipoproteins tend to be retained in blood circulation for a longer time, thereby promoting atherosclerosis. Finally, some lipoprotein remnants containing abundant TG may enter the arterial walls, leading to occurrence and development of atherosclerosis [7, 26]. Therefore, compared with LDL-C, serum non-HDL-C plays a higher effect in inducing atherosclerosis and LDL-C alone tend to neglect the promoting effect of other lipoproteins on ischemic stroke.

This study also showed that cognitive function scores were associated with the type of cerebral stroke in patients with high serum non-HDL-C levels and cerebral infarction. The score was the lowest in patients with CACI/PACI and the highest in patients with LACI, indicating an association with injury to specific brain regions. CACI/PACI mainly involve the middle cerebral artery territory; a large lesion in the dominant hemisphere or bilateral hemispheric lesions can obviously involve the temporal lobe, insula, corpus callosum, hippocampus, and other parts that are closely associated with cognitive and memory functions, whereas the LACI normally has a smaller effect on the cognitive function unless the above specific locations are involved [27].

Cross-sectional analyses revealed that elevation of serum non-HDL-C levels were accompanied by elevated blood glucose and serum HCY levels. Previous studies found that patients with type 2 diabetes often suffered from dyslipidemia, especially patients with uncontrolled blood glucose. Abnormal lipid metabolism is mainly associated with diabetic vascular diseases, where specific lipid modulation or low-fat diet is likely to prevent or delay the progression of diabetic vascular diseases, suggesting that lipid metabolism disorders and diabetes have common pathophysiology for cognitive disorders after cerebral stroke [28]. In addition, patients with diabetes often also suffer from impaired renal function, and it has been shown that patients with decreased flomerular filtration rate also had greater levels of cognitive impairment [29]. Nevertheless, since the relationships of all these factors are complex, additional studies are necessary to assess comprehensively all these factors together.

In this study, regression analyses showed that the risk of cognitive disorders in patients with high serum HCY level was 1.057 times that of patients with a low level. which might be associated with high HCY-induced neurotoxicity [30]. These results agree with a recent study that showed that patients with vascular cognitive impairment have high HCY levels [31]. Accordingly, decreasing HCY levels seems to be associated with better cognitive outcomes in patients with Alzheimer’s disease [32], suggesting that lowering HCY levels after an ischemic stroke could improve the outcomes, but additional studies are necessary to address this issue.

In addition, a low education level, age, high HAMD score, history of cerebral stroke, and family history of dementia were independent risk factors of cognitive disorders after ischemic stroke. These results are consistent with a previous study that showed that lower education, history of diabetes, high HAMD scores, high hsCRP levels, and high HbA1c levels were associated with cognitive impairment after acute ischemic stroke [33]. However, these results were all obtained in relatively small studies and need to be confirmed in larger trials.

This study is not without limitations. This was a single-center retrospective cohort study with a small sample size and limited follow-up duration. Only a limited number of variables could be assessed because of the retrospective nature of the study. Additional studies are necessary to confirm these findings.

## Conclusions

In conclusion, high serum non-HDL-C levels might significantly increase the risk of cognitive disorders after acute cerebral stroke. As an inexpensive and easily measured biomarker, non-HDL-C screening could be undertaken for primary prevention of cognitive disorders after acute cerebral stroke in Chinese adults, but also provide a new direction for investigating treatment for cognitive disorders after acute cerebral stroke.

## Abbreviations

ADL: Activity of Daily Living Scale
BMI: Body Mass Index
HADM-21: Hamilton Depression Rating Scale 21-Item
HbA1c: Glycated Hemoglobin
HCY: Homocysteine
HDL-C: High-Density Lipoprotein Cholesterol
LACI: Lacunar Circulation Infarction
LDL-C: Low-Density Lipoprotein Cholesterol
MMSE: Mini-Mental State Examination
MoCA: Montreal Cognitive Assessment
non-HDL-C: Non-High-Density Lipoprotein Cholesterol
NPI: Neuropsychiatric Inventory
PACI: Partial Anterior Circulation Infarction
POCI: Posterior Circulation Infarction
S-CRP: Serum High-Sensitivity C-Reactive Protein
TACI: Total Anterior Circulation Infarction
TC: Total Cholesterol
TG: Triglycerides
